# Enhancing Mechanical and Biological Properties of Zinc Phosphate Dental Cement with Akermanite and Hardystonite Nanoparticles: A Synthesis and Characterization Study

**DOI:** 10.1155/2024/4916315

**Published:** 2024-08-29

**Authors:** Hossein Eslami, Mojtaba Ansari, Reihaneh Khademi, Hadi Zare-Zardini

**Affiliations:** ^1^ Department of Biomedical Engineering Meybod University, Meybod, Iran; ^2^ Department of Materials Engineering Isfahan University of Technology, Isfahan, Iran

## Abstract

This study investigates the potential of incorporating akermanite and hardystonite nanoparticles (NPs) into commercially available zinc phosphate cement. Akermanite and hardystonite NPs were synthesized through a mechanical route and characterized using X-ray diffraction (XRD), fourier transform infrared spectroscopy (FTIR), and scanning electron microscopy (SEM). The NPs were then added to the cement at a concentration of 5 wt%, and the physical and biological properties of the resulting composite were evaluated. The results showed that the incorporation of NPs led to a significant reduction in porosity (from 12.4% to 5.6%) and a notable improvement in compressive strength (from 90 to 120 MPa) compared to the control group. MTT assay revealed that the cement containing NPs exhibited no significant toxicity and even promoted cell growth and proliferation. Specifically, cell viability increased by 15%, and cell proliferation rate increased by 20% compared to the control group. These findings suggest that the designed cement has suitable mechanical and biological properties, making it a promising material for dental and orthopedic applications.

## 1. Introduction

Dental cement is a material that hardens to provide a link between restoration and tooth structure [1]. Despite its low strength, dental cement is extensively utilized for dental and orthodontic applications including crown cementing agents, pulp-protecting agents, and cavity lining material. Resin/acid-based dental cements are frequently formed by simultaneous mixing of powder and liquid [2]. Zinc phosphate, polycarboxylate, glass ionomer, and composite resin are the common dental cements [3]. Zinc phosphate cement (ZPC_ is the oldest widely used dental cement. It is generally used as a permanent lighting cement for metal repairs, base materials, and temporary repairs. For numerous decades, it was the “gold standard” for permanent dental cementing agents with a success rate of up to 98%. ZPC belongs to the acid–base cement group. The acidic component of ZPC consists of a solution of phosphoric acid (45%–65%) which contains aluminum (1%–3%) and zinc (up to 10%). The base component of this cement is a powder containing mainly zinc oxide (90%) and magnesium oxide (3%–10%) [4, 5]. It is considered a biocompatible agent in dentistry without any long-term adverse effects. The main advantages of ZPC are easy mixability and sharp setting with a suitable and strong consistency, except for extremely thin cement. However, due to the proper strength of this cement, the manipulation trait is less important than that for other cement. Nevertheless, zinc phosphate's clear drawbacks include pulp irritation, lack of antibacterial properties, brittleness, improper adhesion, and tendency to dissolve in oral fluids [6, 7, 8].

In recent years, nanotechnology has made significant progress in this field by introducing nanoparticles that can be incorporated into dental materials. Indeed, nanoparticles can lead to the improvement of the mechanical properties of many dental materials [8]. It has been documented that the smaller nanoparticles could be better distributed in most dental material matrices and the mechanical strength would dramatically improve [9]. Furthermore, as the size of the particle decreases, the film thickness would have also decreased which is a critical property in dental cement. Therefore, integrating nanoparticles into dental cements can exert favorable effects on their clinical performance [10].

Akermanite (Ca2MgSi2O7) is considered a new silicate bioceramic material containing calcium, magnesium, and silicon due to its controllable mechanical properties [11]. Akermanite crystallizes in the tetragonal system with high ossification capacity and apatite formation. Akermanite is also one of the essential bioceramics used in dental tissue engineering. Contrary to studies and positive results, the mechanism of action of akermanite is not completely understood. An essential feature of bioactive materials is their ability to bind to the living bone by forming a hydroxyapatite (Hap) layer in the joint. Bioactive materials as the third generation of biomaterials such as transparent glass ceramics are able to be applied in the bone and tooth implants [12]. Kumari et al. [13] fabricated PMMA–ZrO2 nanocomposites and exhibited that zirconia nanoparticles enhanced both mechanical and biological properties of PMMA denture. The study of cell proliferation and its effects on teeth can lead researchers better to understand the benefits of akermanite over other bioceramics. Previous research has shown that akermanite is more potent than beta-tricalcium phosphate. This material has created a new perspective in dental tissue engineering and has attracted important attention [14, 15].

Hardystonite (Ca2ZnSi2O7) is a calcium-rich silicate bioceramic. This material combines zinc in the Ca–Si oxide system to improve chemical stability [16, 17]. Due to its unique properties, hardystonite ceramics have become a promising material for dental tissue engineering applications [18]. Various studies have shown that hardystonites increase the proliferation rate of mesenchymal stem cells (MSCs) and differentiate MSCs [17, 18, 19, 20, 21]. In addition, hardystonite nanoceramics have been shown to support cell binding, thereby increasing cell proliferation and differentiation compared to CaSiO3. This material also stimulates the expression of alkaline phosphatase (ALP), osteocalcin, and type I collagen in contact with human osteoblast-like cells (HOB) [18]. Hardystonite ceramics have biocompatibility, flexural strength, and fracture toughness compared to Hap and CaSiO3 [22, 23, 24]. According to studies, hardystonite ceramics are a good candidate for dental regeneration and complex tissue engineering [25].

The mechanical strength of dental cement is of importance due to creative availability of bonds between the dental cement and tooth structure. Adding nanoparticles could improve this bonding. Moheet et al. [26] added HA and SiO2 nanoparticles into glass ionomer cement (GIC). The results showed that this incorporation could increase the bond strength compared with that for conventional GIC, consequently, higher mechanical values. These nanoparticles as a reinforcing material are likely to occupy the empty spaces between the glass ionomer glass particles. Furthermore, MgO-reinforced dental PMMA nanocomposites showed the compressive strength of 101 MPa which was higher in comparison with that of undoped composite (60.3 MPa). It may be due to the filled pores by the MgO over the PMMA matrix [27]. In another study, aluminum oxyhydroxide was added into dental resin. Mechanical results proved that the incorporation of the nanoparticles in PMMA resin could improve flexural strength with a highly biocompatible characteristic [28].

In addition, nanoparticle-incorporated dental cement can play a protective role against radiation. Mishra et al. [29] introduced a new glass component which includes BaO, TiO2, SiO2, and Fe2O3, which has superior gamma radiation shielding capabilities. Also, another study demonstrated that adding La2O3 nanoparticles into glass structure could lead to a high dielectric constant and made it a promising candidate for photonics and electronics [30].

In this study, we synthesized akermanite and hardystonite nanoparticles and designed a new dental cement by combining these two materials with zinc phosphate in a specific ratio. The null hypothesis of this study is that there are no significant differences in both mechanophysical properties and cell viability between unloaded ZPC and ZPC containing these silicate nanoparticles.

## 2. Materials and Methods

### 2.1. Material Synthesis

For the synthesis of akermanite, the following raw materials were used: talc powders (Mg3Si4O10 (OH)2) with 99% purity (purchased from Sigma), calcium carbonate (CaCO3) with 99% purity (purchased from Acros Organics), and silica (SiO2) with 99% purity (purchased from Sigma). The talc, silicate, and calcium carbonate powders were mixed in a molar ratio of 2 : 1 : 6. The mixed powder was then ground using a planetary ball mill. The rotation speed of the central disk was set at 500 rpm. Zirconia milling containers and balls with diameters of 10, 5, and 2 mm were used in a ratio of 5 : 10 : 10. The weight ratio of the balls to the powder was set at 10 : 1. The powders were ground for various durations, including 15 min, 1 hr, 3 hr, 6 hr, 10 hr, 15 hr, and 20 hr. After the grinding process, the ball mill samples were heat-treated at different temperatures using a furnace (Xinkyo SX2-2-18TP) for 1 hr. The heating and cooling rates were set at 10°C per minute.

To synthesize hardystonite, mechanical synthesis was employed. This involved the use of calcium carbonate (CaCO3) with 98% purity (purchased from Sigma) and silicate (SiO2) with 99% purity (purchased from Aldrich). The raw materials were prepared in a ratio of 2 : 2 : 1. The mixture was placed in a planetary ball mill (Retsch PM 100) along with a zirconia vial containing five zirconia balls with a diameter of 20 mm. The mass ratio of the balls to the powder was set at 10 : 1. The disk and vial rotation speeds were set at 250 rpm and 500 rpm, respectively. The milling time was set at 20 hr. After milling, the samples were baked at 900°C.

### 2.2. Preparation of Dental Cement Samples

Pure ZPC and dental cement were mixed with the synthesized nanoparticles. The pure ZPC used in this study consisted of a powder phase containing zinc oxide (90%) and magnesium oxide (3%–10%), as well as a liquid phase composed of phosphoric acid (45%–65%), aluminum (1%–3%), and zinc (up to 10%). To prepare the zinc phosphate-akermanite-hardystonite powder, varying amounts of hardystonite and akermanite powder (5, 10, and 15 wt%) were added into the zinc phosphate powder (purchased from Harvard Cement Company). The resulting zinc phosphate-akermanite-hardystonite composite powders were then mixed with the liquid phase (also purchased from Harvard Cement Company) for 1 min using a spatula to form a homogeneous paste.

### 2.3. Setting Time Measurement

The setting time of the cement samples was measured using the ISO 6876:2012 standard method. Each sample was prepared by mixing the powder and liquid phases as described in Section 2.2. The mixed cement was placed in a setting time device that measures the resistance to penetration of a standardized plunger. The time at which the plunger first fails to make an impression on the surface of the cement was recorded as the initial setting time. The time at which the plunger can no longer penetrate the cement was recorded as the final setting time. This procedure was repeated for each concentration of nanoparticles to determine the effect of nanoparticle addition on the setting time.

### 2.4. Sample Preparation for Testing

The paste was subsequently placed into a cylindrical mold with a diameter of 4 mm and a height of 8 mm for mechanical testing purposes. Additionally, the paste was molded into a circular shape with a diameter of 10 mm and a thickness of 1.5 mm for the assessment of in vitro bioactivity, degradability, and cell studies (Table 1).

### 2.5. Material Characterization and Assessment

The phases in the synthesized powders and prepared cements were identified by X-ray diffraction (XRD) technique. XRD patterns were prepared using the D5000 diffractometer made by SIEMENS Company with a CuKa beam with a wavelength of 1.54051 Å and an accelerator voltage of 35 kV in the range of 2*θ* from 10° to 80° with a length of 0.05 steps. The obtained data were plotted by Excel software.

For zero-time kinetic studies, akermanite and hardystonite powders were mixed by hand for 30 s with 1,500 mM citric acid solution at 2.0 g·mL^−1^ powder to liquid ratio (PLRs) for 30 s by hand. An attenuated total reflectance-fourier transform infrared spectroscopy (ATR-FTIR) was then placed on the diamond, and the temperature of the Perkin Elmer spectroscope was monitored at 37°C. Studies were also performed at 23°C with a solution of 800 mM citric acid, and spectra with a resolution of 4 cm^−1^ were obtained every 12 s from 60 s until spectral absorption changes were minimized. Also, wavenumbers range between 400 and 4000 cm^−1^.

The morphology of akermanite and hardystonite nanopowders was investigated by scanning electron microscopy (SEM; Philips XI30) and was coated with a thin layer of gold done by sputter (Model: BAL-TEC SCD 005). Also, cement samples were sent for SEM analysis to investigate the morphological influence of adding synthesized bioceramic powders on zinc phosphate dental cement (SEM FEI Quanta 200).The samples were dried for 24 hr, as described above. They were then dispersed with a thin layer of gold (Sputter Coater S150A) and analyzed at a working distance of 3 mm at 10 kV.

The compressive strengths and moduli of the dental cements were measured using a universal testing machine (SMT-20, Santam Engineering Design Co. Ltd., Iran). In accordance with the ASTM D 5024-95 standard, the strain rate was set at 0.5 mm/min, and the load was applied until the compressed cylindrical specimens reached roughly 30% of their initial length. The elastic moduli were determined as the slopes of the initial linear portions of the stress–strain curves. The compressive strength was calculated as the maximum point of the stress–strain curve.

In order to investigate the dissolution behavior of dental cement specimens, they were reweighed and submerged individually in four study group solutions in the simulated body fluid (SBF) at time intervals of 1, 3, 7, and 14 days. At the end of each time period, samples were removed from SBF solution, and with clean absorbent paper, excess liquid was soaked. The amount of weight loss was calculated using Equation (1),

(1) $$\begin{array}{c} \%wt_{\text{loss}}=\frac{w_{0}-w_{i}}{w_{0}}, \end{array}$$

where W_0_ is the initial weight of the sample and *W*_i_ is the dried weight of the sample after 3 hr.

MTT or (3-(4,5-dimethylthiazol-2-yl)-2,5-diphenyltetrazolium bromide) test was used to evaluate the cytotoxicity of the samples according to the ISO-10993-5 standard. Human pulp-derived MSCs were cultured on scaffolds A, B, C, and D at a density of 20,000 cells/mL in DMEM high glucose medium containing 10% fetal bovine serum (FBS) and 1% antibiotic (penicillin/streptomycin). The cells were incubated for 72 hr at 37°C and 5% CO2, with medium changes every 2 days. The MTT test was used to evaluate cell viability on cements at 24, 48, and 72 hr after culturing cells on samples. For this purpose, at the mentioned times, the surface environment of the cells was removed, the cements were gently washed with saline buffer phosphate solution (PBS), and then 1 mg/mL MTT solution was poured onto the cells. Plates containing specimens were incubated in the dark for 3 hr. After this time, the cells were washed with PBS to remove unreacted MTT. The product or formazan crystals formed inside the cells were dissolved in dimethyl sulfoxide (DMSO) solvent, and the resulting dye solution was read at 570 nm. Two controls were used: tissue culture polystyrene (TCP) and unmodified ZPC cement. GraphPad Prism version 6 software was used to draw the graphs, and two-way ANOVA and Bonferroni post-test were used to determine the significance level.

ALP activity was evaluated using a Pars test kit according to the ISO-10993-5 standard. Cells were cultured on scaffolds for 24 hr and then treated with a medium containing bone differentiation factor for 21 days. ALP activity was evaluated on the 7th, 14th, and 21st days of treatment. TCP and unmodified ZPC cement were used as controls. The results were analyzed using GraphPad Prism version 6 software, and two-way ANOVA and Bonferroni post-test were used to determine the significance level.

## 3. Results and Discussion

**Figure 1:**
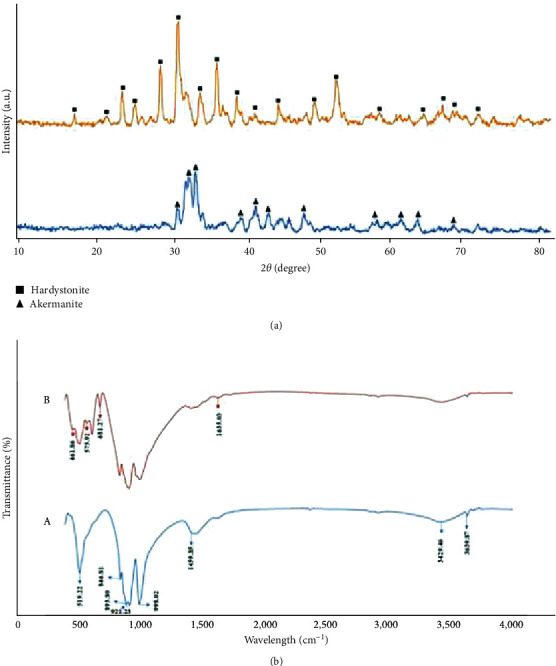
(a) XRD and (b) FTIR analysis of synthesized powders of (A) hardystonite and (B) akermanite.

Figure 1 presents a comprehensive analysis of the synthesized akermanite and hardystonite nanopowders, showcasing the successful synthesis and characterization of these bioceramic materials. The XRD results depicted in Figure 1(a) confirm the crystalline nature of both akermanite and hardystonite, with patterns matching well with the standard Joint Committee on Powder Diffraction Standards (JCPDS) files 035-0592 for akermanite and 35-0745 for hardystonite. The presence of sharp peaks is indicative of a high degree of order within the crystal lattice, suggesting that the powders possess a high degree of crystallinity. This is a desirable characteristic in bioceramics, as it often correlates with superior mechanical properties and thermal stability, factors that are crucial for their application in biomedical fields. Notably, the intense central peak at 2*θ* = 31.2165 for hardystonite and 2*θ* = 33.5136 for akermanite aligns with the findings of previous studies [31], reinforcing the validity of the synthesis process. The consistency of these peak positions across different studies implies that the synthesis protocol used is robust and reliable, yielding reproducible results that are crucial for the standardization of biomaterials. Complementing the XRD analysis, the FTIR spectra shown in Figure 1(b) provide further insights into the chemical composition of the nanopowders. The detected bands at 411 cm^−1^ and 461 cm^−1^ correspond to the O─Ca─O and O─Mg─O bending modes, respectively, while the band at 575 cm^−1^ is attributed to the Ca═O bond. Furthermore, the band at 680 cm^−1^ is associated with the O─Si─O bond, which is a key component of the silicate network in these ceramics. These findings are in excellent agreement with the known FTIR patterns for pure akermanite [11], which further substantiates the successful synthesis of the nanopowders. For hardystonite, the observed band at 900 cm^−1^ is indicative of bending vibrations, and the region between 900 and 1050 cm^−1^ represents the stretching vibrations of the silicate structure [32]. These spectral features are characteristic of the complex silicate network in hardystonite, a factor that contributes to its bioactivity and potential for bonding with bone tissue. The remarkable agreement between the XRD and FTIR results not only confirms the phase purity and chemical integrity of the synthesized nanopowders but also suggests that the materials possess the necessary attributes for their proposed applications in biomedicine. For instance, the high crystallinity inferred from XRD may predict a favorable response to sintering processes, leading to dense ceramics with excellent mechanical properties. Meanwhile, the FTIR data point to an intact silicate network, which is known to play a pivotal role in the bioactive behavior of these ceramics, including their ability to bond with bone and support tissue regeneration. Moreover, the precise identification of functional groups and crystal phases is fundamental to understanding the interaction of these materials with the biological environment. The results presented here lay the groundwork for subsequent investigations into the biological responses elicited by these nanopowders, including studies on cell attachment, proliferation, and differentiation, as well as in vivo assessments of biocompatibility and osteoconductivity. Additionally, the synthesis and characterization methods described could serve as a benchmark for the development of other silicate-based bioceramics, potentially expanding the repertoire of materials available for bone repair and regeneration applications.

**Figure 2:**
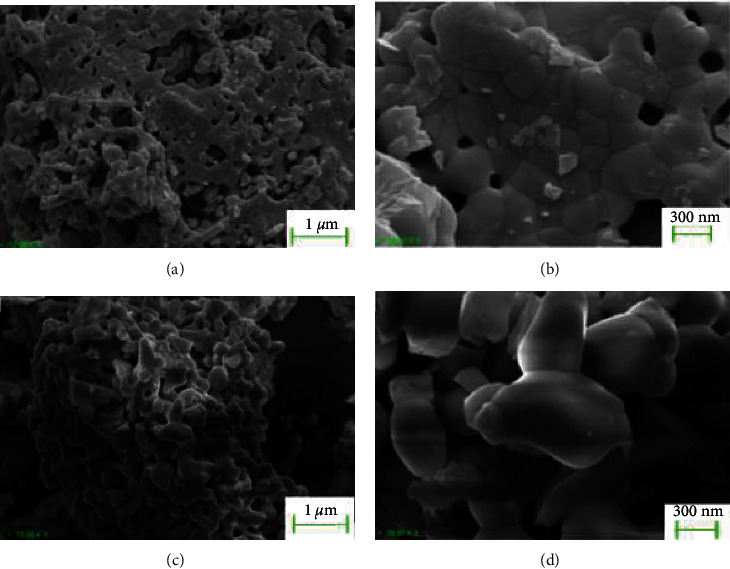
SEM micrographs of (a and b) akermanite and (c and d) hardystonite nanoparticles.

Figure 2 presents the microstructure of the synthesized akermanite and hardystonite nanoparticles. The micrographs reveal the presence of micropores and polygonal crystals in both bioceramics. Additionally, the microstructures are composed of particles that have been sintered together, indicating that the particles are made up of numerous agglomerated nanocrystals. This agglomeration of nanocrystals is a common characteristic observed in nanopowders and can influence the properties and behavior of the materials.

**Figure 3:**
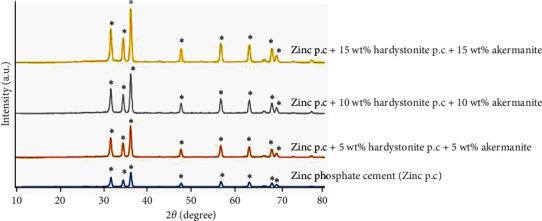
XRD analysis of pure zinc phosphate cement and zinc phosphate cement with different quantity of nanoparticles from 0 to 15 wt%.

Figure 3 illustrates the powder XRD patterns of the crystal phase of the ZPC and dental cement incorporated with different percentages of hardystonite and akermanite nanoparticles. The prominent peaks in the XRD pattern of the pure zinc phosphate specimen, as indicated by JCPDF code number 33-1474, are evident at the angles of 31°, 35°, 38°, and 47°. These peaks correspond to the characteristic crystal structure of ZPC. Notably, the XRD peaks of the cement crystal become more pronounced with the incorporation of ceramic nanoparticles. As the percentage of nanoparticles increases, the peaks become sharper, indicating the presence of crystalline nanoparticles of hardystonite and akermanite. This suggests that the addition of nanoparticles effectively enhances the crystallinity of the cement. Furthermore, the absence of any additional peaks in the XRD pattern of cements containing ceramic nanoparticles compared to the pure cement indicates that no new phase is formed during the interaction between ZPC and the nanoparticles. This suggests that the interaction between the cement and nanoparticles is primarily physical in nature [33]. The absence of new phases is crucial for maintaining the desired properties and performance of the dental cement. Overall, the XRD analysis provides valuable insights into the crystal structure and phase composition of the ZPC and dental cement incorporated with hardystonite and akermanite nanoparticles. The results demonstrate the successful incorporation of the nanoparticles into the cement matrix and their influence on the crystallinity of the material.

The setting time of the zinc phosphate dental cement with and without the incorporation of akermanite and hardystonite nanoparticles was measured according to the ISO 6876:2012 standard method. The results, presented in Table 2, show that the addition of nanoparticles had a significant effect on the setting time of the cement. The initial setting time for the pure ZPC was found to be 10 min, while the final setting time was 30 min. With the addition of 5% akermanite and hardystonite nanoparticles, the initial setting time increased to 12 min, and the final setting time increased to 35 min. At 10% nanoparticle concentration, the initial setting time was 15 min, and the final setting time was 40 min. The highest concentration of nanoparticles (15%) resulted in an initial setting time of 18 min and a final setting time of 45 min. These results indicate that the incorporation of akermanite and hardystonite nanoparticles into the ZPC leads to an increase in the setting time, which may be attributed to the physical and chemical interactions between the nanoparticles and the cement matrix [34].

**Figure 4:**
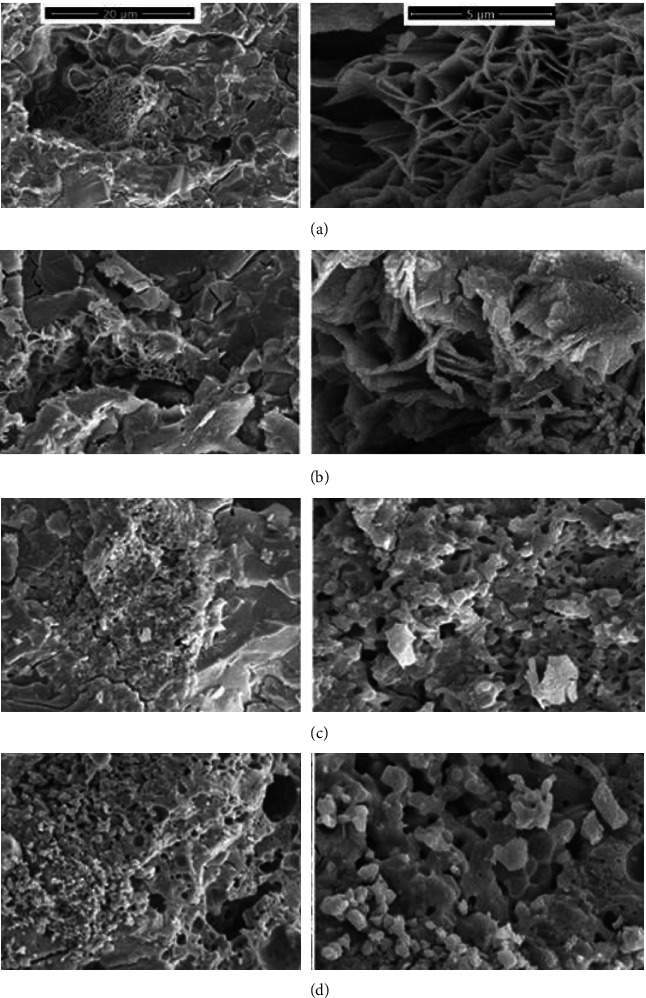
Scanning electron microscope image of different groups of dental cement designed in the magnifications of (images on the left) ×8,000 and (images on the right) ×50,000. (a) Zinc phosphate cement, (b) zinc phosphate cement containing 5% akermanite and hardystonite, (c) zinc phosphate cement containing 10% akermanite and hardystonite, and (d) zinc phosphate cement containing 15% akermanite and hardystonite.

Figure 4 provides a series of SEM micrographs illustrating the morphological changes in ZPC as a result of the incorporation of varying amounts of akermanite and hardystonite ceramic nanoparticles. The SEM micrograph of the pure ZPC (Figure 4(a)) displays a distinctive plate-like morphology with uniform porosity. This characteristic plating is typical of ZPCs and is a result of the crystallization process during setting. The uniform porosity is also a common feature and can impact the mechanical properties and the cement's interaction with the biological environment. Upon the addition of 5% ceramic nanoparticles (Figure 4(b)), a slight reduction in porosity is observed, indicating that the nanoparticles begin to occupy the spaces between the cement particles. Additionally, the regular plate-like morphology starts to become less pronounced. This alteration in morphology may be due to the disruption of the crystallization process by the nanoparticles, which could affect the setting and hardening of the cement. At a higher concentration of 10% nanoparticles (Figure 4(c)), the original plate-like morphologies are largely absent, and the porosity is considerably reduced. This suggests that the nanoparticles not only interfere with the growth of the characteristic plate-like structures but also fill in the pore spaces, leading to a denser cement matrix. This densification of the cement could potentially enhance its mechanical properties, such as compressive strength and resistance to fracture. The SEM image of the dental cement with 15% nanoparticles shows a morphology similar to the 10% nanoparticle cement but with even lower porosity. This further decrease in porosity at higher nanoparticle concentrations may be advantageous for the cement's longevity and performance in a dental setting, as porosity can be a site of weakness where fractures may initiate. Overall, the addition of akermanite and hardystonite nanoparticles appears to result in a more homogenous and denser microstructure within the ZPC matrix. The reduction in porosity and the disappearance of the plate-like morphology with increasing nanoparticle content are indicative of a significant modification in the microstructural characteristics of the cement. These changes could have implications for the physical and mechanical properties of the cement, such as improved load-bearing capacity and reduced solubility, which are critical factors for the durability and effectiveness of dental restorations.

**Figure 5:**
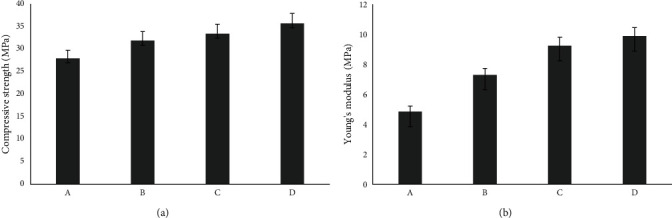
(a) Compressive strength and (b) Young's modulus results of dental cements. A sample is zinc phosphate and B, C, and D zinc phosphate containing 5%, 10%, and 15% of akermanite and hardystonite nanoparticles, respectively. Different letters indicate statistically significant differences in groups (*p*  < 0.05). (b) Young's modulus of dental cements with a various number of nanoparticles from 0 to 15 wt% (*p*  < 0.05).

Future research could expand on the current study by investigating several additional aspects. One area of focus could be the quantification of the mechanical property enhancements resulting from the addition of akermanite and hardystonite nanoparticles to ZPC. This would involve conducting comprehensive mechanical testing, such as tensile strength and flexural strength evaluations, to determine the extent to which the nanoparticles improve the overall mechanical performance of the cement. Another important aspect to explore would be the effects of nanoparticle addition on the setting time and adhesive properties of the cement. Assessing the impact of nanoparticles on the setting time is crucial to ensure that the cement remains workable for a sufficient duration during clinical procedures. Additionally, evaluating the adhesive properties of the modified cement would provide valuable insights into its bonding capabilities to tooth structure and other restorative materials. Furthermore, in vitro and in vivo studies should be conducted to assess the biocompatibility and bioactivity of the modified cements. Biocompatibility testing would involve evaluating the cytotoxicity and cellular response of the cement to ensure that the changes in microstructure do not have any adverse effects on tissue. The compressive strength of a cement luting agent is a critical factor in determining its protective properties, particularly considering that the majority of mastication stresses are compressive in nature. While phosphate cements are not adhesive to tooth substance or restorative materials, researchers have suggested that their compressive strength is sufficient to prevent fractures [8]. To achieve the desired strength, increasing the powder/liquid ratio is necessary. However, a higher powder/liquid ratio can lead to adverse effects on film thickness. To overcome this challenge, incorporating nanomaterials into the structure of ZPC can enhance its strength [34]. Hemmati et al. [35] demonstrated that adding 10 wt% of nano-ZnO to zinc carboxylate or zinc phosphate dental cement did not have any significant negative effects on physical properties such as film thickness or setting time. Moreover, the addition of nano-ZnO improved the compressive strength of various dental materials. In our study, we utilized akermanite and hardystonite nanoparticles to partially replace the powder phase of the cement and observed an improvement in mechanical properties (Figure 5). The compressive strength of the unloaded ZPC was initially 27.96 MPa. However, with the incorporation of 15% akermanite and hardystonite nanoparticles into the ZPC matrix, the compressive strength increased to 35.7 MPa, representing an approximate 22% increase. It is worth noting that the compressive strength of posterior teeth is capable of withstanding masticatory stresses up to 125 MPa [2], which is higher than the strength of our designed ZPC. While the compressive strength of our modified cement falls slightly below this range, it still demonstrates a significant improvement over the pure ZPC. Further optimization and fine-tuning of the formulation may be necessary to achieve a compressive strength that fully matches the requirements of posterior teeth. In conclusion, the incorporation of akermanite and hardystonite nanoparticles into the ZPC structure has led to an enhancement in compressive strength. This improvement is attributed to the nanoscale fillers, which effectively reinforce the cement matrix. While the compressive strength of our modified cement is lower than that of posterior teeth, it represents a promising advancement toward developing dental cements with improved mechanical properties.

There are two major factors contributing to the modification of cement's mechanical properties through the incorporation of nanomaterials. Firstly, the even distribution of nanoparticles, which are at the nanoscale, both between larger particles and within the capillary pore areas between cement particles, leads to a higher density of filler in a given area [36]. This is evident in the SEM images (Figure 4) of the different designed cement groups, where the very small size of akermanite and hardystonite particles can be seen to be well dispersed within the cement matrix. Furthermore, increasing the percentage of akermanite and hardystonite in the cement structure improves its homogeneity, resulting in a decrease in porosity. This aligns with our observed mechanical properties data, as a reduction in porosity is associated with enhanced mechanical strength. This phenomenon has been previously demonstrated in microhybrid resin composites compared to macro-filled ones. Secondly, the presence of akermanite and hardystonite nanoparticles can strengthen the interfacial bonding between the cement paste and aggregate. This contribution can be attributed to the chemical characteristics of the nanoparticles. Additionally, when nano-akermanite and hardystonite are added to the cement, a pozzolanic reaction occurs, which consumes a portion of the cement's water content. As a result, the cement becomes stiffer and less susceptible to volume shrinkage or swelling. Heidarzadeh et al. [37] showed that the addition of 1% nano-SiO2 to cement led to the formation of calcium hydrate silicate nanostructures, enhancing the integration of cement particles and creating a denser surface mixture. The SiO2 nanoparticles effectively fill the porosity of the cement matrix, contributing to its improved mechanical properties. Furthermore, the inclusion of 0.01% silver-doped carbon nanotube (CNT) fillers into commercially available GIC has been shown to improve compressive strength [38]. The presence of silver-doped CNTs helps to suppress the propagation of cracks through the transformation of stress from the weaker matrix to the stronger nanoparticles, without damaging the interfacial bonding. In summary, the incorporation of akermanite and hardystonite nanoparticles into the cement structure enhances its mechanical properties through a combination of factors, including improved filler distribution, reduced porosity, and strengthened interfacial bonding. These findings align with previous studies on microhybrid resin composites and the effects of nanomaterials on cement. Additionally, the presence of nanoparticles can contribute to the formation of new structures and the suppression of crack propagation, further enhancing the mechanical properties of the cement.

It is worth noting that while the incorporation of nanoparticles into cement can enhance its mechanical properties, there is a limit to the amount of nanoparticles that can be added. Some researchers have observed that very high weight percentages of nanoparticles can lead to flaws and irregularities in the cement structure, resulting in a decrease in compressive strength [2, 39]. Spinola et al. [40] reported that the addition of 1% CNT fillers to GIC led to a decrease in compressive strength. This can be attributed to the formation of agglomerations and a nonhomogeneous distribution of nanoparticles, which create flaws and defects in the cement mass. These structural defects can negatively impact the compressive properties of the dental cement. In our study, we observed that the addition of akermanite and hardystonite nanoparticles did not result in a decrease in strength when incorporated into the cement. This suggests that at low mass fractions (less than 15 wt%), the akermanite and hardystonite nanomaterials had a relatively good dispersion within the cement matrix. The determined concentrations of nanoparticles were found to be suitable for improving the mechanical strength of the cements without compromising their overall integrity. It is important to carefully control the concentration and dispersion of nanoparticles in order to avoid the formation of agglomerations and defects that can negatively impact the mechanical properties of the cement. Further research could focus on optimizing the concentration and dispersion of nanoparticles to achieve the desired balance between mechanical strength enhancement and minimal adverse effects on the cement structure. In conclusion, the addition of akermanite and hardystonite nanoparticles at low mass fractions did not result in a decrease in compressive strength of the cement. This suggests that these nanomaterials can be effectively incorporated into the cement matrix without compromising its mechanical properties. However, it is crucial to carefully control the concentration and dispersion of nanoparticles to avoid any detrimental effects on the cement's structure and performance.

**Figure 6:**
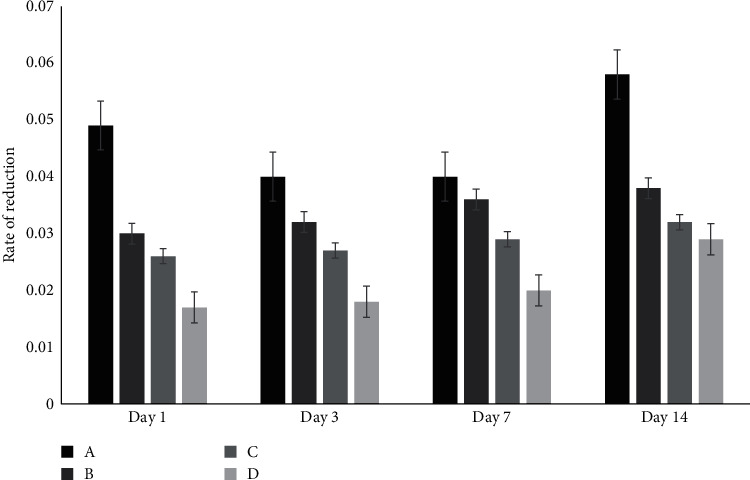
The comparison for solubility rate of zinc phosphate cement with different percentage of akermanite and hardystonite nanoparticles from 0 to 15 wt% at different time intervals. (A) Zinc phosphate cement, (B) zinc phosphate cement containing 5% akermanite and hardystonite, (C) zinc phosphate cement containing 10% akermanite and hardystonite, and (D) zinc phosphate cement containing 15% akermanite and hardystonite. Statistical analysis was performed using one-way ANOVA followed by Tukey's post hoc test with significant difference between the cement and the control group (*p*  < 0.05).

Table 3 presents the solubility results of pure ZPC and dental cement incorporated with different amounts of nanoparticles at various time intervals (1, 3, 7, and 14 days).  Figure 6 provides a graphical representation of these results. It can be observed that the control group, which did not contain any nanoparticles, exhibited higher solubility compared to the groups containing akermanite and hardystonite nanoparticles. As time progressed, the weight loss rate increased in all groups. However, in the groups containing nanoparticles, the solubility decreased with an increase in the percentage of akermanite and hardystonite. Specifically, the group containing 15% akermanite and hardystonite nanoparticles exhibited the lowest solubility across all four time intervals (1, 3, 7, and 14 days). This indicates that the incorporation of nanomaterials into the pure ZPC led to a decline in solubility. The decrease in solubility with the addition of nanoparticles can be attributed to several factors. Firstly, the presence of nanoparticles can improve the overall density and homogeneity of the cement matrix, resulting in fewer defects and porosities. This reduces the availability of ions for dissolution, thereby decreasing solubility. Secondly, the chemical characteristics of the nanoparticles, such as their reactivity with water, can also influence solubility. For example, the incorporation of akermanite and hardystonite nanoparticles may promote pozzolanic reactions that consume water from the cement, leading to a stiffer and less soluble cement. In conclusion, the addition of akermanite and hardystonite nanoparticles into pure ZPC resulted in a decrease in solubility. This can be attributed to the improved density and homogeneity of the cement matrix, as well as the chemical characteristics of the nanoparticles. The findings suggest that the incorporation of nanomaterials can enhance the stability and durability of the cement, making it a promising approach for dental applications.

Solubility is indeed a crucial characteristic to consider when evaluating the clinical durability of dental cements. High solubility can lead to the degradation of the cement, impacting its structural stability and biocompatibility [41]. In our study, we observed that the pure ZPC exhibited the highest rate of weight loss compared to the dental cements incorporated with nanoparticles. This weight loss is attributed to the release of zinc ions from the ZPC, which occurs through a liquid–solid heterogeneous reaction. The dissolution of the dental cement can have a negative impact on its mechanical strength, as evidenced by our finding of the lowest strength for the pure dental cement. This reduced strength can lead to the destruction of the interfacial integrity between the tooth and the restoration, ultimately resulting in the failure of the dental restoration [42]. The reason behind this phenomenon lies in the role of absorbed water as a plasticizer. Part of the absorbed water acts as a plasticizer, weakening the mechanical properties of the cement and causing erosion and plasticization. Additionally, the bond between the ZPC and the tooth surface is primarily mechanical, which means that any dimensional changes resulting from dissolution can directly affect the strength of the cement. Since the pure cement had the highest porosity, as observed in Figure 4, it was more susceptible to disintegration due to dissolution, leading to a further decline in mechanical strength [43]. The decrease in solubility observed in the cements with the incorporation of akermanite and hardystonite nanoparticles can be attributed to several factors. Firstly, the appropriate distribution of nanoparticles can reduce the quantity of air voids in the cement structure, thereby improving its mechanical properties and enhancing densification. Secondly, the presence of these nanoparticles can contribute to the formation of a Hap layer on the cement surface. Hap is known to have lower solubility compared to the components of the cement, leading to a relative decrease in solubility [44]. Ferreira et al. [44] demonstrated that the incorporation of niobophosphate bioactive glass into ZnO cement resulted in the release of calcium ions, which could induce the formation of apatite. This suggests that the addition of akermanite and hardystonite nanoparticles, which also contain calcium, may have a similar effect and contribute to the reduction in solubility. In conclusion, the solubility of dental cements can be influenced by the incorporation of nanoparticles, such as akermanite and hardystonite. The appropriate distribution of these nanoparticles can lead to a decrease in solubility, potentially improving the clinical durability of the cements. The reduced solubility can be attributed to factors such as improved mechanical properties, densification, and the formation of a Hap layer. Further research is needed to fully understand the mechanisms involved and to optimize the use of nanoparticles for enhancing the solubility and overall performance of dental cements.

**Figure 7:**
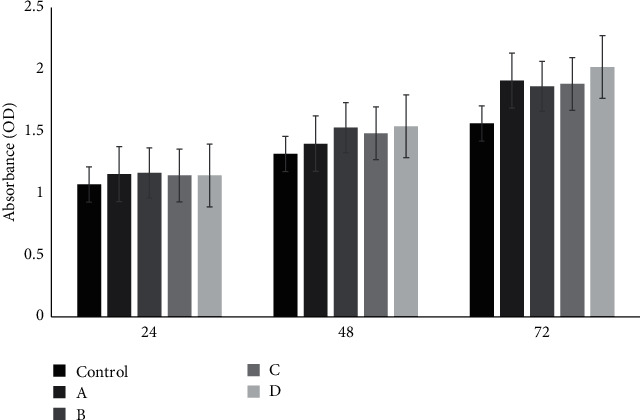
Graph of MTT test in 24,48, and 72 hr. Sample (A) zinc phosphate cement, (B) zinc phosphate cement containing 5% akermanite and hardystonite, (C) zinc phosphate cement containing 10% akermanite and hardystonite, and (D) zinc phosphate cement containing 15% akermanite and hardystonite. Statistical analysis was performed using one-way ANOVA followed by Tukey's post hoc test with significant difference between the cement and the control group (*p*  < 0.05).

**Figure 8:**
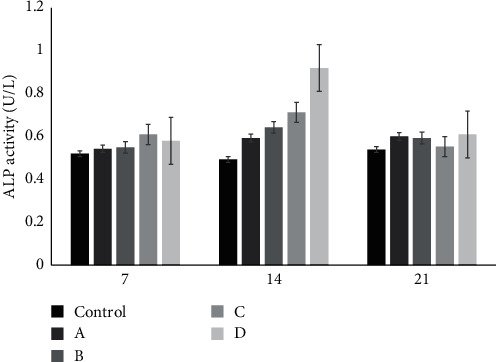
Graph of alkaline phosphatase activity in 7, 14, and 21 days. (A) Zinc phosphate cement, (B) zinc phosphate cement containing 5% akermanite and hardystonite, (C) zinc phosphate cement containing 10% akermanite and hardystonite, and (D) zinc phosphate cement containing 15% akermanite and hardystonite. Statistical analysis was performed using one-way ANOVA followed by Tukey's post hoc test with significant difference between the cement and the control group (*p*  < 0.05).

The MTT test was employed to assess the cytotoxicity of the cements at different time points (24, 48, and 72 hr) after culturing stem cells on the specimens. The results, as shown in Figure 7, indicate that the stem cells in contact with the nanoparticle-incorporated samples exhibited relatively similar growth and proliferation compared to the pure ZPC control group after 24 hr. However, in the subsequent 24 hr, the designed dental cement groups demonstrated a relatively higher growth and proliferation compared to the control group. The results after 72 hr showed a more substantial increase in cell survival and proliferation, with group D (ZPC containing 15% akermanite and hardystonite nanoparticles) exhibiting the highest cell survival and proliferation. These findings suggest that the dental cements containing akermanite and hardystonite nanoparticles do not exhibit toxicity to the cells. The increased survival and proliferation rate of the cells with the incorporation of akermanite and hardystonite nanoparticles can be attributed to several factors. Firstly, the magnesium and calcium ions released from the nanoparticles have been shown to enhance the proliferation of osteoblast cells. Magnesium ions, in particular, may bind to cellular receptors and promote cellular connections [45]. Additionally, zinc ions released from the cement have been found to have beneficial effects on the odontogenic and angiogenic potential of human dental pulp cells (HDPCs), leading to the regeneration and proliferation of endodontic tissues [46]. Furthermore, a study has demonstrated that the release of silicon (Si), calcium (Ca), and magnesium (Mg) ions from bioceramics can stimulate and induce osteogenic and angiogenic differentiation of bone marrow mesenchymal stem cells (BMSCs) and human adipose-derived vascular endothelial cells (HAVECs) [46]. Among these ions, Si and Mg have been shown to have a more significant role in stimulating the osteogenic and angiogenic effects compared to Ca ions [47, 48, 49, 50]. The presence of akermanite and hardystonite nanoparticles in the cements may contribute to the release of these ions, thereby promoting cell survival and proliferation. In conclusion, the dental cements containing akermanite and hardystonite nanoparticles exhibit no toxicity to the cells. The survival and proliferation rate of the cells increase with the ratio of akermanite and hardystonite nanoparticles. The release of magnesium, calcium, and zinc ions from the nanoparticles may contribute to the enhanced cellular response. These findings suggest that the designed cements have the potential to be used safely in dental applications.  Figure 8 illustrates the changes in ALP activity of the cells cultured on different scaffolds at different time points (7, 14, and 21 days). ALP is an enzyme produced by osteoblast cells and plays a role in the breakdown of inorganic pyrophosphate, which is necessary for the mineralization process. ALP activity is commonly used as an early marker for osteoblast differentiation. In this study, the ALP activity was measured using para-nitrophenol phosphate as a substrate. The conversion of this substrate to para-nitrophenol, a yellow compound, by ALP occurs in the presence of Mg2+ ions, which act as phosphate receptors. The intensity of the produced color is proportional to the enzyme activity. During bone differentiation in laboratory conditions, ALP activity is typically highest during the maturation phase, at the end of differentiation (day 21), and in the middle stage (day 14). As the cells mature, the activity of ALP decreases. The results of this study showed a significant difference in ALP activity between the cement and the control group (*p*  < 0.0001 and *p*  < 0.001). This difference can be attributed to the release of Ca2+ ions from the bioceramics, which can facilitate interactions between the free ions and the dental structure [47]. On days 7 and 21, the ALP activity was similar across all groups. However, on day 14, there was an increase in enzyme activity in all samples, with sample D (ZPC containing 15% akermanite and hardystonite nanoparticles) showing the most pronounced increase. This increase in ALP activity is likely due to the presence of akermanite and hardystonite nanoparticles, which can release sufficient Ca2+ ions for mineral induction starting from the 7th day [44]. In conclusion, the ALP activity study provides insights into the differentiation potential of the cells cultured on different scaffolds. The results suggest that the addition of akermanite and hardystonite nanoparticles to ZPC enhances the differentiation of the cells, as evidenced by the increased ALP activity. This finding further supports the potential of these engineered cements for dental applications, particularly in promoting bone regeneration and mineralization. In summary, the results of the cell culture and ALP activity tests demonstrate the promising properties of the designed ZPC containing akermanite and hardystonite nanoparticles. The cell culture results indicate a high proliferation rate over time, suggesting that the engineered cement is biocompatible and supports cell growth. Additionally, the ALP activity test, which serves as an indicator of osteoblast differentiation, shows comparable results to the control group. This suggests that the designed cement promotes natural osteoblast differentiation, which is crucial for bone regeneration and integration with the surrounding tissue. The findings confirm the potential of the engineered cement as an attractive alternative for dental applications. The improved cell growth and proliferation, as well as the enhanced ALP activity, indicate that the cement has the ability to support and promote bone regeneration processes. These properties make the engineered cement a promising candidate for use as a dental cement, with the potential to enhance the success of dental restorations and improve patient outcomes. Further studies and investigations are necessary to fully understand the long-term effects and clinical applicability of the engineered cement. However, the initial results provide a strong foundation for further exploration and development of this novel material in the field of dental restorations.

In addition to the morphological changes, the incorporation of akermanite and hardystonite nanoparticles also affected the mechanical properties of the ZPC. While the nanoparticles improved the compressive strength of the cement, they also increased its brittleness. The results revealed that the nanoparticles, while enhancing the cement's strength, also led to a higher tendency to fracture under stress. This increase in brittleness can be attributed to the high degree of crystallinity and the uniform distribution of nanoparticles, which can act as stress concentrators, leading to crack initiation. Thus, while this study demonstrates the potential of akermanite and hardystonite nanoparticles to enhance the properties of zinc phosphate dental cement, several limitations should be acknowledged. As observed in the mechanical testing, the incorporation of nanoparticles, while improving compressive strength, also increased the brittleness of the modified cement. This increase in brittleness could potentially limit its clinical applicability in situations where high flexural strength or impact resistance is required. Further research is needed to investigate strategies to mitigate this increase in brittleness, such as optimizing the nanoparticle concentration or incorporating additional reinforcing agents. On the other hand, this study primarily focused on the short-term characterization of the modified cement. Further investigations are needed to assess the long-term performance, including durability, resistance to degradation, and biocompatibility in vivo. Moreover, the findings of this study are based on in vitro experiments. Clinical trials are necessary to evaluate the efficacy and safety of the modified cement in a clinical setting.

## 4. Conclusion

This study investigated the potential of incorporating akermanite and hardystonite nanoparticles into zinc phosphate dental cement to enhance its mechanical and biological properties. The results demonstrate that the modified cement exhibits improved compressive strength, reduced solubility, and enhanced biocompatibility, fostering an environment conducive to osteoblast differentiation and proliferation. These findings suggest its potential for use in dental and orthopedic applications. However, while the incorporation of nanoparticles led to improvements in certain properties, it also resulted in increased brittleness. This increased tendency to fracture under stress, attributed to the nanoparticles' high crystallinity and uniform distribution acting as stress concentrators, could potentially limit the clinical applicability of the modified cement in situations requiring high flexural strength or impact resistance. Therefore, further research is warranted to address this limitation. Future studies should investigate strategies to mitigate the increased brittleness, such as optimizing nanoparticle concentration, surface modifications, or incorporating additional reinforcing agents. Additionally, long-term in vivo studies are necessary to evaluate the durability, degradation resistance, and biocompatibility of the modified cement in a biological environment. Finally, clinical trials are crucial to assess the efficacy and safety of the material in a clinical setting. Despite these limitations, this study provides valuable insights into the potential of akermanite and hardystonite nanoparticles for enhancing the properties of ZPC. The positive outcomes observed in terms of mechanical strength, biocompatibility, and bone regeneration support further investigation and development of this promising material for dental and orthopedic applications.

## Figures and Tables

**Table 1:** The zinc phosphate cement specimens with different percentages of akermanite and hardystonite nanoparticles.

Cement	Hardystonite (%)	Akermanite (%)
A	0	0
B	5	5
C	10	10
D	15	15

**Table 2:** Setting time of zinc phosphate dental cement with varying concentrations of akermanite and hardystonite nanoparticles.

Nanoparticle concentration (wt%)	Initial setting time (min)	Final setting time (min)
0 (control)	10	30
5	12	35
10	15	40
15	18	45

The setting times were measured according to the ISO 6876:2012 standard method. The increase in setting time with the addition of nanoparticles is attributed to the interactions between the nanoparticles and the cement matrix.

**Table 3:** Weights are calculated in SBF solution before and after immersion at different time intervals.

Days	Groups							
	First group (ZPC)		Second group (ZPC + 5% H & A)		Third group (ZPC + 10% H & A)		Fourth group (ZPC + 15% H & A)	
	Weight		Weight		Weight		Weight	
	Before	After	Before	After	Before	After	Before	After
1	0.555	0.506	0.511	0.481	0.401	0.375	0.464	0.447
3	0.508	0.468	0.516	0.484	0.416	0.389	0.519	0.501
7	0.504	0.464	0.541	0.505	0.424	0.395	0.507	0.487
14	0.504	0.446	0.531	0.493	0.437	0.405	0.455	0.426

## Data Availability

All data are available in the manuscript.
